# Comparison of slow and fast neocortical neuron migration using a new *in vitro *model

**DOI:** 10.1186/1471-2202-9-50

**Published:** 2008-06-05

**Authors:** Anna J Nichols, Laurel H Carney, Eric C Olson

**Affiliations:** 1 Department of Neuroscience and Physiology, SUNY Upstate Medical University, Syracuse, NY 13210, USA; 2 Department of Biomedical Engineering, University of Rochester, Rochester, NY 14642, USA; 3 Department of Neurobiology & Anatomy, School of Medicine and Dentistry, University of Rochester, Rochester, NY 14642, USA

## Abstract

**Background:**

Mutations, toxic insults and radiation exposure are known to slow or arrest the migration of cortical neurons, in most cases by unknown mechanisms. The movement of migrating neurons is saltatory, reflecting the intermittent movement of the nucleus (nucleokinesis) within the confines of the plasma membrane. Each nucleokinetic movement is analogous to a step. Thus, average migration speed could be reduced by lowering step frequency and/or step distance.

**Results:**

To assess the kinetic features of cortical neuron migration we developed a cell culture system that supports fiber-guided migration. In this system, the majority of fiber-apposed cells were neurons, expressed age-appropriate cortical-layer specific markers and migrated during a 30 min imaging period. Comparison of the slowest and fastest quartiles of cells revealed a 5-fold difference in average speed. The major determinant of average speed in slower cells (6–26 μm/hr) was step frequency, while step distance was the critical determinant of average speed in faster cells (>26 μm/hr). Surprisingly, step distance was largely determined by the average duration of the step, rather than the speed of nucleokinesis during the step, which differed by only 1.3-fold between the slowest and fastest quartiles.

**Conclusion:**

Saltatory event frequency and duration, not nucleokinetic speed, are the major determinants of average migration speed in healthy neurons. Alteration of either saltatory event frequency or duration should be considered along with nucleokinetic abnormalities as possible contributors to pathological conditions.

## Background

The architecture of the cerebral cortex is established by the outward migration of neurons from the proliferative ventricular zone to their assembling cellular layer within the cortical plate. Disruption of migration by genetic mutations or gestational exposure to toxins can cause a number of neurological disorders including epilepsy, mental retardation and learning disorders such as dyslexia [1]. Although cortical neurons exhibit multiple distinct modes of migration [2-4], a dominant mode is saltatory migration along either radial glial fibers [5] or axonal fibers [6,7].

Despite the identification of genes and biochemical interactions essential for neuronal migration [8], basic questions about the nature of the movement itself remain unanswered. Among these: what distinguishes a fast from a slow moving cell? Do cells change migration rate by varying the speed of nucleokinesis, the frequency of movements, or the distance traveled per movement? These questions are most directly addressed *in vitro *where the intrinsic elements of movement can be observed with fewer constraints imposed by surrounding tissue and where pharmacological dissection of the frequency and amplitude components of movement is possible. In this report we describe a novel *in vitro *system for investigating neocortical migration that employs a standard preparation of embryonic cortical neurons. This system has allowed us to quantify the kinetic features that distinguish slow and fast moving cortical neurons.

## Results

### Cellular clustering and the formation of fiber fascicles *in vitro*

**Figure 1:**
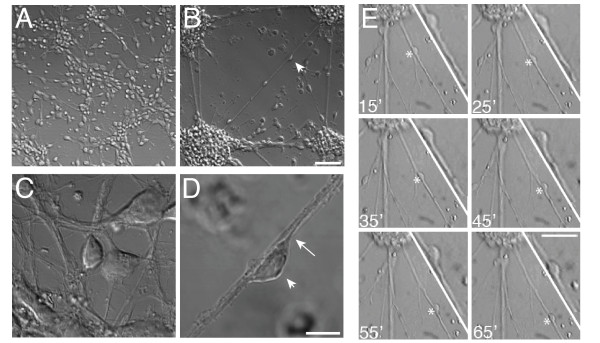
Formation of lattice cultures. **A**) DIC images of E17 cultures before and **B**) 1 DIV after Serum Free Medium (SFM) addition. **C, D**) Higher magnification (arrowhead in B and D identifies the same cell). **D**) Fiber-apposed cells have an elongated soma and a distinct leading process (arrow). **E**) Transmitted light time-lapse imaging reveals that fiber-apposed cells are mobile. A migrating cell is indicated in each 10 min image series (asterisk). Insets are digitally magnified. For a time-lapse movie of cell movement see Additional file 1. Scale bars: (**A, B**) 50 μm, (**C, D**) 10 μm, (**E**) 30 μm.

Embryonic cortical neurons lose motility when dissociated and plated on common tissue culture substrates and this has hampered the development of *in vitro *models of neuronal migration. This lack of motility may be due in part to strong cellular adhesion on non-permissive substrates. We found that the addition of small amounts of anti-adhesive cell suspension medium (293 Serum Free Medium) to fully dissociated cultures of embryonic cortical neurons (Fig. 1A,C) at 1 day *in vitro *(1 DIV) caused the aggregation of cells into clusters interconnected by a lattice of fiber fascicles at 2 DIV (Fig. 1B,D). After aggregation, cells that had an elongated somal morphology characteristic of migrating neurons were observed on fibers (Fig. 1D). Time-lapse imaging confirmed that many of the apposed cells migrated along the fiber fascicles (Fig. 1E and see Additional file 1).

**Figure 2:**
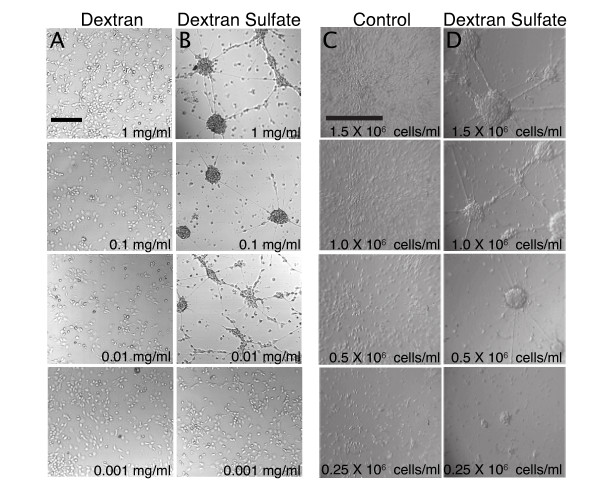
Dextran sulfate causes lattice culture formation. Bath application of 5 kD dextran sulfate, but not 5 kD dextran causes lattice culture formation in **A-B**) concentration-dependent and **C-D**) cell density-dependent manners. Scale bars: 400 μm.

To identify components of 293 Serum Free Medium that provide clustering activity, standard cortical cultures were prepared from embryonic day 15 (E15) neocortices. Dorsal neocortices were dissected, dissociated in Trypsin-EDTA and plated in 96 well plates. Time-lapse studies used tissue culture plastic plates that were pretreated with 25 μg/ml poly-D-lysine (PDL) while immunocytochemical studies used glass-bottomed plates that were pretreated with 50 μg/ml PDL. No differences in clustering were observed between PDL-treated plastic and PDL-treated glass plates. The cultures were allowed to develop for 1 DIV prior to assaying clustering activity. Amicon size-exclusion centrifugation revealed that the active fraction of 293 SFM was 5–100 kD. The active fraction could not be heat inactivated (95°C, 2 hr) and was insensitive to trypsin digestion (37°C, 4 hr). Soluble dextran sulfates (DS) are anti-adhesive agents that are often included in cell suspension media. We found that addition of 5 kD DS at 1 DIV similarly induced lattices by 2 DIV at concentrations ranging from 10–100 μg/ml (Fig. 2B). Dextran (5 kD) alone was unable to induce clustering, highlighting the importance of the negatively charged sulfate moiety (Fig. 2A). In contrast, other components of SFM including the non-ionic detergent Pluronic did not cause appreciable clustering.

Although DS induced clustering, the formation of fiber lattices depended on both cell density and the presence of neurites in culture. When DS (100 μg/ml) was added at the time of dissociation and plating, before cells extended neurites, lattices did not form. Smaller nodes and longer lattices were observed with decreasing cell density, and clusters but not fiber lattices were observed when cell density dropped below 0.5 × 10^6 ^cells/ml (Fig. 2D).

### Composition of lattice cultures

**Figure 3:**
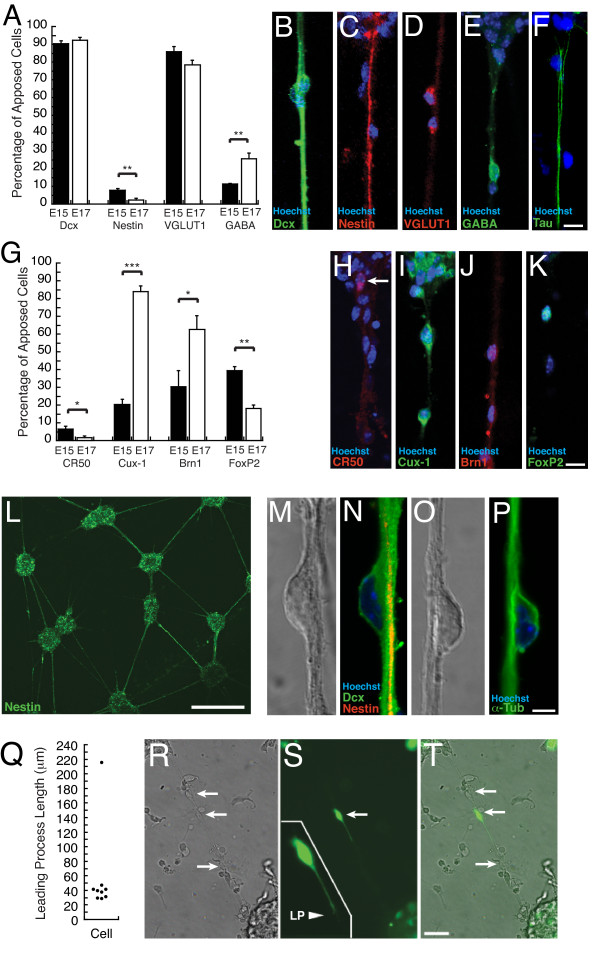
Characterization of lattice cultures. **A**) Apposed cells are immunopositive for the neuronal marker doublecortin (Dcx) and a marker of excitatory neurons, VGLUT1 at E15 and E17. E17 cultures contained significantly more GABA expressing apposed cells and fewer nestin expressing apposed cells. **B-F**) Images from E17 cultures. **G**) Apposed cells in E15 and E17 cultures differ in the expression of layer specific cellular markers, CR50+ (layer 1), Cux-1+ (layer 2–4), Brn1+ (layer 2–5), FoxP2+ (layer 5–6). **H-K**) Images from E17 cultures. **L**) Nestin immunoreactivity of nodes and fiber fascicles in an E17 culture. **M**) DIC image of **N**) a Dcx+ (green) neuron apposed to a nestin+ fiber (red). Hoechst nuclear stain is blue. **O**) DIC image of an apposed cell **P**) α-tubulin immunolocalization reveals the characteristic tubulin cage (green) encasing the nucleus (blue). **Q-T**) Morphology of migrating neurons. **Q**) Leading process (LP) length at E17 in apposed cells transfected with eGFP expressing Sindbis virus; average length was 57.3 ± 6.7 μm (n = 9). **R**) Transmitted light and **S**) fluorescence images of apposed neurons (arrows). Inset is digitally magnified and reveals 3 filopodia at the end of the LP (arrowhead). **T**) Overlay. Scale bars: (**F, K**) 10 μm, (**L**) 500 μm, (**P**) 5 μm, (**T**) 20 μm. Immunocytochemical quantification was performed on a minimum of 200 cells from 3 separate cultures at E15 and E17. Analyses of fiber fascicles was performed on a minimum of 40 fascicles from 2 separate experiments at E15 and E17. Student's t-test values: *, p = 0.05; **, p = 0.01; ***, p < 0.001.

To determine the identities of cells apposed to fibers, lattice cultures were immunostained for cell type specific markers at E15 and E17, 2 DIV after plating at either E13 or E15, respectively (Fig. 3). Lattice cultures are susceptible to "washout" during immunochemical solution exchanges, presumably due to reduced adhesion caused by DS addition. To stabilize the lattice network prior to immunostaining, 5 μl of a 2% solution of calfskin gelatin (Aldrich) was gently added to each well and allowed to solidify. The cultures were then treated using a standard immunocytochemical protocol (see Methods). Doublecortin (Dcx) is a microtubule associated protein (MAP) expressed by migratory neurons in the developing cortex [9] and the majority (>90%) of the observed fiber-apposed cells were positive for Dcx (Fig. 3A,B). Greater than 78% of apposed cells at both time points were immunopositive for VGLUT1 (Fig. 3A,D), a vesicular glutamate transporter that identifies excitatory neurons [10]. In contrast, 11% of apposed cells in E15, and 25% in E17 cultures, were immunopositive for GABA (Fig. 3A,E; p = 0.01). A small fraction was positive for the neuronal precursor marker nestin, while no apposed cells expressed the glial marker GFAP (data not shown). Thus the fiber-apposed cell class at E15 and E17 was primarily excitatory neurons.

Since laminar identity in the cortex is assigned prior to migration, apposed cells were immunolabeled for layer specific markers (Fig. 3G–K). Cux-1 [11] identifies later born upper-layer cortical neurons and immunolabeled 20% of the apposed cells at E15, and 84% at E17 (p = 0.001). In contrast, FoxP2, a marker of deep cortical layers [12], identified 40% of apposed cells at E15 and 20% at E17 (p = 0.01). At both culture time points the anti-Reelin antibody CR50, which identifies Cajal-Retzius cells in layer 1 [13], labeled few apposed cells. Thus, the fiber-apposed class of cells was largely reflective of the migrating class of neurons at the corresponding embryonic day *in vivo*.

The composition of fiber fascicles in lattice cultures was examined in cultures immunolabeled for nestin and RC2, markers for radial glial cells and their processes [14,15], Dcx, a marker for immature cortical neurons and their neurites, and tau, a microtubule-associated protein that is highly enriched in axons. The majority of fiber fascicles displayed individual fibers that were immunoreactive for nestin (Fig. 3C,L) and RC2 (data not shown). Nestin expression was detected in 72% of fiber fascicles at E15, and 59% of fascicles at E17. All (100%) of fiber fascicles were strongly immunopositive for Dcx at both E15 and E17, while most fiber fascicles were immunopositive for the axonal marker tau (Fig. 3F). Eighty seven percent of fascicles at E15, and 84% of fascicles at E17 contained tau expressing fibers. These results demonstrate that the interconnecting fibers were a mixture of radial glial and neuronal processes. The fiber composition of lattice cultures indicates potential support of axonal-guided [6] and glial-guided [5] migration.

To determine the morphological features of migrating neurons in lattice cultures, cells were immunolabeled for either Dcx (Fig. 3N) or α-tubulin (Fig. 3P) along with the nuclear stain Hoechst. Apposed cells displayed a perinuclear cage of Dcx and α-tubulin characteristic of migrating cortical neurons [9,16]. Cultured cells were labeled with eGFP expressing Sindbis virus [17] and fiber-apposed cells were examined (Fig. 3Q–T). Apposed cells exhibited a short trailing process and an extended leading process (57.3 ± 6.7 μm; n = 9) that was consistent with the 30–50 μm leading process length observed in cortical slice culture [2].

**Figure 4:**
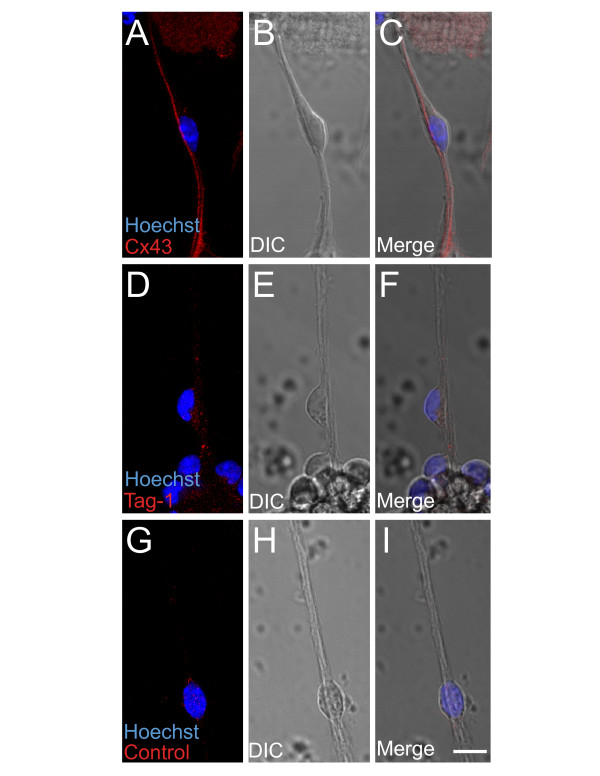
Expression of neuronal adhesion proteins in lattice cultures. **A-C**) Connexin 43 (Cx43) is expressed by neurons and fiber fascicles in lattice cultures whereas **D-F**) Tag-1 is not detected. **G-I**) Control immunolabeling with the Alexa Fluor 555 secondary antibody alone. Scale bar: 10 μm.

A number of adhesion molecules including connexin 43 (Cx43), connexin 26 (Cx26) [28,30] and Tag-1 [6,32,33] have been implicated in neuronal migration and positioning. Targeted deletion or acute suppression of Cx43 alters excitatory neuron migration patterns into the developing cortical plate [28,30], while the adhesion molecule Tag-1 is expressed by corticofugal fibers and may be involved in interneuron migration into the cortex [6,32,33]. Lattice cultures show strong immunolabeling for Cx43 (Fig. 4A–C) but little or no Tag-1 immunoreactivity (Fig. 4D–F). The expression pattern of Cx43 is consistent with a role for Cx43 in mediating the adhesion of migrating neurons in lattice cultures.

### Migration kinetics of apposed cells

**Figure 5:**
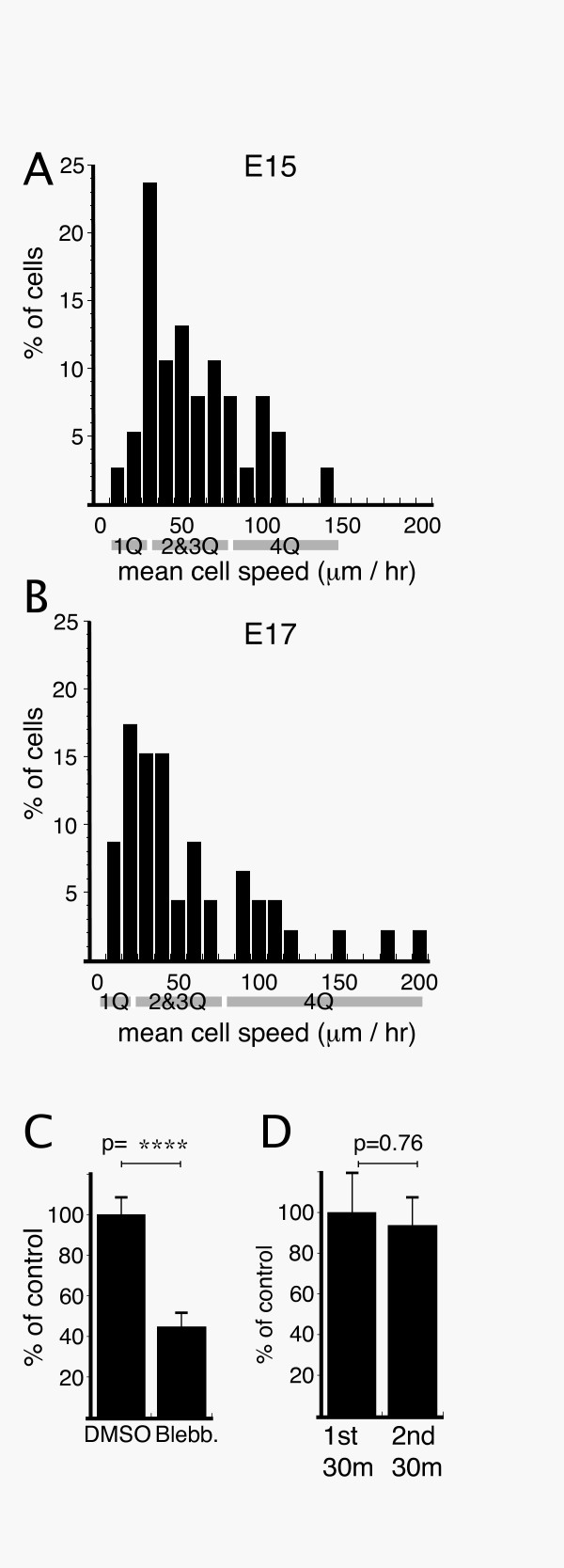
Characterization of migration in lattice cultures. Distribution of mean cell speeds during 30 min observations in **A**) E15 and **B**) E17 cultures. **C**) Inhibition of myosin-II with 100 μM blebbistatin significantly reduced migration speed compared to DMSO control. **D**) Mean migration speed was the same in the second 30 min of transmitted light illumination compared to the first 30 min. A minimum of 35 cells from 3 separate cultures were analyzed for each experiment (A-D). Quartiles are assigned from median values (median values of total, upper 50% and lower 50% of the mean cell speed distribution) in A and B. 1Q = 1st Quartile. Student's t-test value: ****, p < 0.0001.

To characterize the migration of fiber-apposed neurons, time-lapse microscopy was performed on E15 and E17 cultures. To ensure optimal cell viability and imaging conditions, the imaging microscope was placed into a tissue culture incubator and allowed to thermally equilibrate overnight. Transmitted light images were recorded the following day using a Logitech Webcam ported to one of the microscope ocular tubes. This configuration provided 640 × 480 pixel images and allowed for extended imaging periods of 16 hrs without evidence of phototoxicity. Images were acquired at 2–5 min intervals and speeds were determined by measuring the cumulative displacement during the first 30 min of imaging. At E15 and E17, 14.8% and 21.1% of the apposed cells, respectively, did not move within a 30 min period. Moving cells had an average speed of 53 ± 5 μm/hr (n = 38) at E15 (Fig. 5A) and 47 ± 6 μm/hr (n = 35) at E17 (Fig. 5B), indicating that the two populations have the same intrinsic speed (p = 0.49). Although DS is required to initiate clustering of cells and fasciculation of processes, we have found that it is apparently not required to sustain the observed migration on fibers. Lattice cultures were induced with a 3 hr application of 100 μg/ml DS, and then the DS-containing media was replaced with normal media. One day after DS application and removal, lattice cultures formed and time-lapse imaging showed that apposed cells migrated at 46 ± 5 μm/hr (n = 32) the same rate measured for cells in the presence of DS (p = 0.89).

To determine if cortical neurons were sensitive to myosin-II inhibition, 100 μM blebbistatin was bath applied to migrating neurons. Blebbistatin caused a 60% decrease in migration speed (Fig. 5C) compared to DMSO control (p < 0.0001), indicating cortical neurons, like migrating interneurons [7,18] depend upon myosin-II activity for movement.

**Figure 6:**
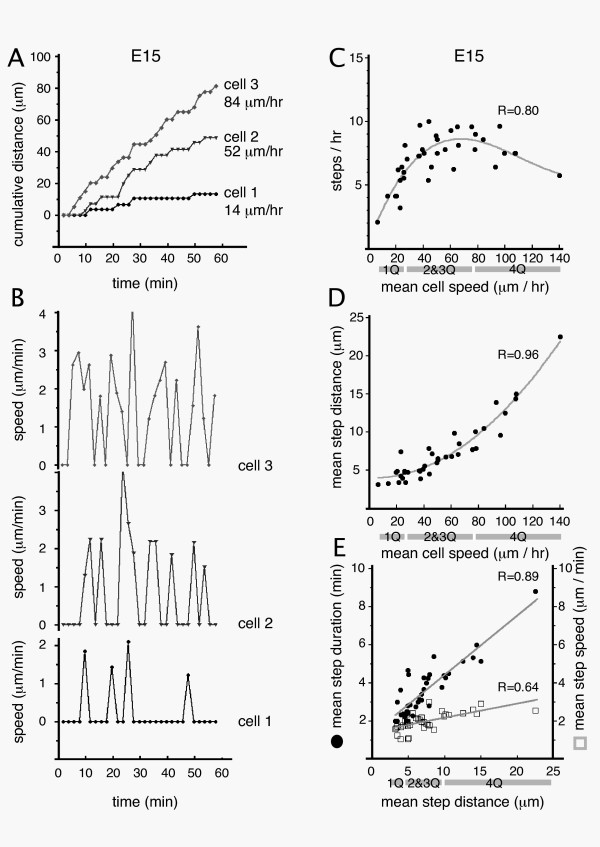
Relation between saltatory movements and mean cell speed in E15 lattice cultures. **A**) Cumulative distance plots from 3 representative cells (of 38 cells analyzed) showing distinct mean speeds. **B**) Speed plots of the same cells as in A, showing differences in the number and size of saltatory movements (steps). **C**) Step frequency and **D**) mean step distance were plotted against the mean speed of the same cell. **E**) Mean step distance compared to mean step duration (closed circles, left Y-axis) and mean step speed (open boxes, right Y-axis) in the same cell. Quartiles are based on mean cell speed (**C, D**) or mean step distance (**E**) and are identified underneath the relevant plots (1Q = 1st quartile).

To assess saltatory migration behavior, E15 cultures were imaged at 2 min intervals for 1 hr. No decrement in mean migration speed was observed in the second 30 min of imaging compared to the first (p = 0.38), suggesting that continued transmitted light illumination for 1 hr is not phototoxic (Fig. 5D). Intervals of somal motility (steps) appear as periods with positive slopes in cumulative distance plots (Fig. 6A). In the corresponding speed plots (Fig. 6B) somal motility appears as discrete peaks separated by valleys where somal speed did not exceed our spatial detection limit (see Methods). The majority of cells (90%) showed a mixture of short- and long-duration saltatory events, indicating that saltatory event duration is not an invariant feature of each cell (Fig. 6B).

We compared the average speed of each cell (n = 38) against step frequency (Fig. 6C) and average step distance (Fig. 6D). The scatter plot indicates a positive relationship between average speed and step frequency in slower cells (6–26 μm/hr) but not in faster cells (>26 μm/hr). While the step frequency plot was poorly fit with a line (Pearson's R = 0.44, not shown), a third order polynomial fit of step frequency (R = 0.80; Fig. 6C) suggests faster cells plateau in step frequency at 7.9 ± 0.2 steps/hr (n = 28 cells in 2Q-4Q). Interestingly, a second order fit of the same data (R = 0.77, not shown) predicts a sharper decline in step frequency in the very fastest cells (>80 μm/hr). This decline could represent the "fusion" of individual steps as the fastest cells approach continuous motility at an estimated ~150 μm/hr. While step frequency differences distinguished the slowest cells (1Q) from faster cells (2Q-4Q), difference in step frequency did not separate cells within the faster group (2Q-4Q). Instead mean step distance (Fig. 6D) separated cells in the faster group (linear fit R = 0.92, 2^nd^-order polynomial fit, R = 0.96). Thus critical determinants of average speed are step frequency in slow cells (6–26 μm/hr), and step distance in faster cells (>26 μm/hr).

The observed increases in step distance in faster cells could be produced by increases in the somal speed attained during the step (step speed) or increases in the temporal duration of the step (step duration) or both (Fig. 6E). While the fastest quartile of cells showed 2.9-fold greater mean step distance than the slowest quartile (12.9 vs. 4.4 μm/step), the mean step speed of the fastest quartile was only 1.3-fold greater than the slowest quartile (2.4 vs. 1.8 μm/min). In comparison, average step duration was 2.2-fold longer in the fastest quartile compared to the slowest (5.3 vs. 2.4 min/step). Thus the 2.9-fold difference in mean step distance between slow and fast cells was produced largely by increased mean step duration, and to a lesser degree by increased mean step speed. This suggests that nucleokinetic speed is not a major determinant of overall migration speed in healthy cells.

To determine whether individual cells exhibited rhythmic saltatory events we used the Fast Fourier Transform (FFT) to extract the frequency components of the speed plots. The FFT extracted a dominant frequency that was, on average, equivalent to the frequency determined by counting saltatory events during a defined period (7.7 ± 1 vs. 7.6 ± 0.3 steps/hr; p = 0.6, n = 38). However, the amount of energy was spread widely across the frequency spectrum and the dominant frequency in individual cell speed plots never exceeded 3% of the total energy at all frequencies. The coefficient of variation (CV) in the peak-to-peak intervals was CV = 0.51 for E15 migratory cells, whereas an idealized rhythmic cell would display a peak-to-peak CV = 0. These analyses suggest that saltatory migration *in vitro *is relatively arrhythmic.

## Discussion

In this study we show that DS addition to dissociated neuronal cultures promotes cellular clustering and the formation of fiber fascicles which serve as substrates for migrating neurons. We use this new cell culture model to identify kinetic distinctions between slow and fast moving cortical neurons.

At present, cortical migration studies rely on *in vivo *analysis and organotypic slices preparations. Improvements in acute cortical slice preparations have permitted detailed analysis of migration movements [2,19], but because the migrating cells are entirely contained within the slice of tissue, the slice preparation may not allow rapid application of some reagents (recombinant proteins, function blocking antibodies and pharmacological reagents) at defined concentration, to the migrating cells. Moreover, the slice preparation does not permit phase contrast and DIC microscopy that are extensively used in other studies of cellular motility.

*In vitro *analysis of neocortical migration, where cells are completely accessible, has primarily relied on one model called the imprint assay [20]. This assay involves cutting 200 μm thick slices of cortex, digesting them briefly with a protease, and then imprinting the slice onto a sticky substrate (Cel-Tak). After washing away the non-adherent cells, a layer of cells is left on the substrate. The layer contains many cells including radial glial cells with attached, migrating neurons. The assay is a useful tool in the analysis of radial glial guided migration and has provided important insights into critical signaling pathways [20-22]. Lattice cultures complement existing models and have a number of strengths including ease of preparation, physiological migration speed and suitability for imaging and pharmacology.

Lattice culture formation is induced by DS addition to the media. Although the mechanism of DS induced conversion of dissociated cortical cultures is not known in detail, the negatively charged DS may neutralize the positively charged PDL substrate thereby reducing cellular adhesion [23]. This reduction in substrate adhesion appears to change the adhesive preference of cells, promoting cell-cell adhesion and allowing cell bodies to aggregate while their processes fasciculate. Similar formations were observed subsequent to lectin treatment of granule cell cultures and were termed 'cable cultures' [24]. Once these fiber networks are established DS does not appear to be required to sustain migration as removal of DS by media exchange does not change migration rate.

Migrating neurons display a polarity, with a distinct leading process, trailing processes and asymmetric localized organelles [5]. The migrating cell's leading process appears to extend and retract in a fashion that does not directly anticipate the forward movement of the nucleus [7,18,25]. Instead a dilation of the basal portion of the migrating neuron's leading process precedes the forward movement of the nucleus. This dilation is enriched in membrane vesicles, microtubules, and the centrosome, a microtubule-organizing center [7,18]. Centrosomal movements appear to precede nuclear translocation [7,18,26,27] and recent work reveals that centrosome advancement is essentially constant in cortical neurons but the nucleus moves in a saltatory fashion into the forming dilation [27].

The average migration rate in lattice cultures is ~1.5-fold higher than the reported migration rate of locomoting (saltatory) neurons in slice explants [2] and 4–5 fold higher than diI (1,1'-dioctadecyl-3,3,3',3'- tetramethylindocarbocyanine perchlorate)-labeled neurons in slice culture [19] or neurons migrating in imprint cultures [20]. However the average migration rate in lattice cultures is the same as that reported for cortical neurons exiting slice explants and migrating into matrigel matrix (49.6 ± 6.6 μm/hr; p = 0.64) [27] indicating that cortical neurons can travel at ~50 μm/hr in the absence of surrounding tissue. The difference in average speed between explants and *in vitro *approaches may reflect simple steric hindrance or a more complicated interplay of adhesion and chemotropic factors.

In our study we find that overall migration speed is correlated with the frequency of saltatory events in slow cells and the amplitude of saltatory events (distance moved) in faster cells. This relationship emerges from the observation that mean step speed (i.e. nucleokinetic speed), in isolation, cannot account for the 5-fold difference in average cellular speed between the slowest and fastest quartiles of cells. While pathological conditions may alter the speed of nuclear movement, the relative constancy of step speed in our study suggests that the molecular mechanisms that move the nucleus are not a primary determinant of average speed in healthy neurons. Average step distance accounted for most (R = 0.96) of the variance between slow and fast cells (Fig. 6D), despite the different classes of migrating neurons (Fig. 3A–K), and the mixed neuronal and glial character of the fascicles (Fig. 3B,C,F). Thus the relationship between overall speed and average step distance may be a fundamental feature of fiber-guided migration. This finding draws attention to the mechanisms that dictate average step distance.

Depending on the cell class (e.g. cerebellar granule vs. cortical neuron), step distance may be determined by the distance between the nucleus and the dilation [7,18,27], or by the distance between the nucleus and the centrosome immediately prior to nucleokinesis [16,26]. If centrosome advancement was continuous rather than saltatory [27], step distance would be limited by the position of dilation formation in the basal leading process. Little is known about the mechanism that triggers the formation of the dilation, although the possibility that the dilation represents a site of cellular adhesion has been proposed [18]. This suggestion outlines a model in which reducing the number of cellular adhesion sites would increase step distance and potentially increase average migration rate. In situations of low cellular adhesion like those thought to occur at the end of migration, cellular movement would be relatively continuous and rapid, as has been observed in terminally translocating cells [2].

Neurons migrating in the radial direction establish a specialized junction with the radial glial fiber termed the "interstitial density." This density is filled with filamentous material and is characterized by a 20 nm dilation between apposed plasma membranes [20]. Although the specific proteins that form this junction are not known, normal radial migration appears to require connexin 43, connexin 26 [28], and IgCAM family member CHL1 [29]. Although neuronal subtype specific migration defects are found in mice lacking CHL1 [29], no single adhesion molecule has been identified which is absolutely required for radial migration *per se*. For example, suppression of connexin 43 [30] impairs, but does not prevent migration through the cortical plate. These findings imply that migrating neurons possess multiple, partially redundant adhesion systems. Similarly, tangential migration of interneurons occurs along axons that express the cell adhesion molecule Tag-1 during early cortical development [32]. Tag-1 has been functionally implicated in tangential migration [6] however, mice deficient in Tag-1 do not show pronounced cortical interneuron migration deficits [33]. Observations of rapid switching between migration modes (e.g. tangential to radial) in organotypic slice cultures [19,34] also suggest the coincident expression of multiple adhesion systems. Given the mixture of axonal and radial glial fibers found within the fiber fascicles and the multiple redundant adhesion systems deployed *in vivo*, it seems likely that migrating neurons in lattice cultures will deploy multiple, functionally redundant classes of adhesion molecules.

The mechanism governing step frequency is unknown. In our study, cells traveling above 26 μm/hr exhibit a step frequency of ~8 steps per hour. However these steps lack rhythm, suggesting that there is not an intrinsic pattern generator underlying step frequency. In migrating cerebellar granule cells, the advancement of the soma is coincident with transient elevations of intracellular Ca^2+ ^and, imposing or blocking these transients alters the rate of migration [35]. If migrating cortical neurons exhibit similar calcium transients during migration, such transients might regulate the saltatory component of migration, namely nucleokinesis, rather than the apparently smooth centrosomal advancement. In this emerging model, step frequency would be related to the frequency of dilation formation in the leading process as well as the frequency of saltatory event initiation.

Recent studies have begun to shed light on the motors underlying nucleokinesis. Insufficiency in the lissencephaly gene Lis1 reduces migration rate [36] by either decoupling the centrosome from the nucleus [26] or preventing the forward advancement of the centrosome and the nucleus [27]. Lis1 appears to regulate the activity of cytoplasmic dynein, a minus-end directed microtubule motor required for centrosomal advance and nucleokinesis [27]. Dynein is found around the nucleus and in the dilation of the basal leading process, suggesting it may exert a "pulling" force on the nucleus. However, dynein is not the only motor involved in nucleokinesis; multiple studies, including our own, have shown that blockade of myosin-II prevents somal advance [7,18,27]. As myosin-II immunoreactivity is found primarily behind the nucleus [7,18], it may exert a "pushing" or protrusive force in concert with dynein's pulling force. The relationship between these two force-generating systems is not completely clear but there is evidence that microtubule destabilization may activate myosin-II to initiate nucleokinesis [18]. Sorting out the mechanisms underlying saltatory movement will require further pharmacological and genetic studies in which fiber-guided migration can be explored quantitatively.

## Conclusion

To analyze fiber-guided migration of cortical neurons, we have developed and characterized a new cell culture model called lattice cultures. Using this approach we found that step frequency and step distance were the major determinants of overall migration rate *in vitro*. In contrast, nucleokinetic speed was relatively constant regardless of overall migration rate. This new culture system provides a simple, quantitative approach to understanding the mechanism(s) of neuronal migration.

## Methods

### Lattice cultures

Swiss Webster mice (Taconic) were cared for by the Department of Laboratory Animal Resources, an AAALAC accredited facility. The animals were treated according to a protocol approved by the IACUC at SUNY Upstate Medical University. Dorsal neocortices were dissected and cortical cultures were prepared at early and mid-corticogenesis, corresponding to embryonic day 13 (E13) or E15 using 0.25% Trypsin-EDTA dissociation. Cells were plated in Neurobasal medium with 2% B27, 1× Glutamax, 1× Penicillin-Streptomycin, and 30 mM glucose (all reagents from Invitrogen) on 96-well plates coated with 25 or 50 μg/ml poly-D-lysine (Sigma). Cultures were maintained in a 37°C, 5% CO_2 _incubator. After 1 day *in vitro *(DIV), lattice cultures were induced with Invitrogen 293 SFM (20% v/v) or 100 μg/ml 5 kD dextran sulfate (MP Biomedicals). 5 kD dextran (ICN Biomedicals) was used in control studies. Cultures prepared on E13 and E15 were used for time-lapse microscopy or immunocytochemistry after 2 DIV, equivalent to E15 and E17.

### Time-lapse imaging

Time-lapse microscopy was performed in a tissue culture incubator using an Olympus IMT-2 microscope with a Logitech Webcam (640 × 480 pixel) mounted in one ocular of the microscope. Logitech software acquired the images.

### Migration analysis

ImageJ v1.34 (Wayne Rasband, NIH) was used to measure the position of the center of the soma in consecutive images and the absolute distance traveled was determined. All cells apposed to fibers were included in our analyses except cells in clusters of 3 or more, cells attached to a significantly deforming fiber, and cells that detached from the fiber. A measurement-detection limit was empirically determined by repeatedly measuring a fixed point in consecutive images. If the absolute distance traveled by a cell between consecutive images was less than 3 standard deviations of our motion-detection limit (3 sd = 2.3 pixels or 1.2 μm at 40× objective magnification), then zero displacement was recorded for that time interval. A saltatory step was defined as any increase in speed surrounded on both sides by periods of zero speed. Step distance refers to the total change in position (regardless of direction) between periods of time with zero displacement. Step duration refers to the total length of time of a saltatory event. Speed (Fig. 5B) refers to the speed attained during the corresponding 2 min image interval. Mean step speed (Fig. 5E) refers to the mean speed attained during all recorded steps for a given cell.

### Immunocytochemistry

Cultures were embedded in 2% calf-skin gelatin (Aldrich) prior to fixation in 4% paraformaldehyde (30 min). Cells were incubated overnight (4°C) with the indicated primary and secondary antibodies diluted in PBS (with 0.1% Triton-X-100, 2% BSA and 5% Donkey serum). Anti-Dcx (1:500, Dr. C. Walsh), anti-nestin (1:10, Developmental Studies Hybridoma Bank, University of Iowa), anti-GABA (1:200, Sigma), anti-VGLUT1 (1:1000, Chemicon), anti-CR50 (1:20, Dr. M. Ogawa), anti-Cux-1 (1:20, Santa Cruz), anti-Brn1 (1:20, Santa Cruz), anti-FoxP2 (1:1000, Abcam), anti-RC2 (1:10, DSHB), anti-tau (1:200 tau-1, Millipore) anti Tag-1 (1:4, clone 3.1C12, DSHB) anti-connexin 43 (1:250, BD Transduction Labs) and anti-α-tubulin (1:1000, Sigma) were used. Appropriate Alexa Fluor 555- and Alexa Fluor 488-conjugated secondary antibodies (Invitrogen) were used at 1:500 dilutions. Nuclei were counterstained with Hoechst 33342 (1 μg/ml).

### Morphology

Medium containing 10^6 ^infectious particles/ml of eGFP expressing Sindbis virus (gift of D. Feldheim, UCSC), was added to cortical cultures at 10–20% (v/v). Cells were examined the following day (E17). Leading process length was measured in 9 eGFP-labeled cells from 2 separate experiments on E17.

### Confocal image acquisition

Images for quantification of marker expression and leading process length were acquired with a Zeiss LSM510 using a 40× lens through a depth of 20–30 μm at 1–2 μm intervals. All cells with individual Hoechst-labeled nuclei apposed to fibers in the flattened z-series projections were counted.

### Analysis and Statistics

Line fitting was performed in Kaleidagraph v3.6.1. Fast Fourier Transform was performed in MATLAB. Significance was tested with the unpaired Student's t-test.

## Authors' contributions

AJN performed the cellular motility assays, immunostaining and quantification. AJN participated in data analyses and manuscript preparation. LHC developed MATLAB software for the analysis of the frequency of saltatory events and participated in data analyses of cellular movement. ECO designed the study, participated in cell culture experiments, data analyses and manuscript preparation. All authors have read and approved the manuscript.

## Supplementary Material

Additional file 1 Migration in lattice cultures. Click here for file
